# AFCo1, a meningococcal B-derived cochleate adjuvant, strongly enhances antibody and T-cell immunity against *Plasmodium falciparum *merozoite surface protein 4 and 5

**DOI:** 10.1186/1475-2875-8-35

**Published:** 2009-02-27

**Authors:** Gustavo Bracho, Caridad Zayas, Lina Wang, Ross Coppel, Oliver Pérez, Nikolai Petrovsky

**Affiliations:** 1 Department of Immunology, Finlay Institute, 17 Ave, Playa, PO Box: 16017, Havana City, Cuba; 2 Department of Microbiology, Monash University, Clayton Vic 3800, Australia; 3 Flinders University and Vaxine Pty Ltd, Bedford Park, Adelaide 5042, Australia

## Abstract

**Background:**

Whilst a large number of malaria antigens are being tested as candidate malaria vaccines, a major barrier to the development of an effective vaccine is the lack of a suitable human adjuvant capable of inducing a strong and long lasting immune response. In this study, the ability of AFCo1, a potent T and B cell adjuvant based on cochleate structures derived from meningococcal B outer membrane proteoliposomes (MBOMP), to boost the immune response against two *Plasmodium falciparum *antigens, merozoite surface protein 4 (MSP4) and 5 (MSP5), was evaluated.

**Methods:**

Complete Freund's adjuvant (CFA), which is able to confer protection against malaria in animal MSP4/5 vaccine challenge models, was used as positive control adjuvant. MSP4 and 5-specific IgG, delayed-type hypersensitivity (DTH), T-cell proliferation, and cytokine production were evaluated in parallel in mice immunized three times intramuscularly with MSP4 or MSP5 incorporated into AFCo1, synthetic cochleate structures, CFA or phosphate buffered saline.

**Results:**

AFCo1 significantly enhanced the IgG and T-cell response against MSP4 and MSP5, with a potency equivalent to CFA, with the response being characterized by both IgG1 and IgG2a isotypes, increased interferon gamma production and a strong DTH response, consistent with the ability of AFCo1 to induce Th1-like immune responses.

**Conclusion:**

Given the proven safety of MBOMP, which is already in use in a licensed human vaccine, AFCo1 could assist the development of human malaria vaccines that require a potent and safe adjuvant.

## Background

Infection with *Plasmodium *parasites is one of the most important health problems of tropical countries, with 500 million clinical cases and over 1 million malaria deaths annually. Notwithstanding that multiple *species *can infect humans; *Plasmodium falciparum *is responsible for the majority of malaria deaths[1]. The development of an effective malaria vaccine remains a critical public health objective given that vector control is not always easy or effective and drug resistant strains are increasingly emerge [2-5]. Although an enormous amount of knowledge on malaria pathogenesis has been accumulated over recent years, no highly protective vaccine has yet emerged. As a result of the improvement of antigen identification and expression technologies, many promising malaria antigens have been cloned and evaluated, but unfortunately this has not translated into clinical success. Proteins exposed on the surface of the asexual blood stage [6], including merozoite surface proteins (MSP)1, MSP2, MSP4, MSP5 and MSP8 represent potential targets to generate asexual blood stage vaccines [2,6-12]. MSP4 and MSP5 are currently in late-stage preclinical development and GMP manufacture in anticipation of human clinical testing . Both proteins are encoded by a tandem region on chromosome 2 of *P. falciparum *and share structural similarities, including a glycosylphosphatidylinositol anchor (GPI) and an epidermal growth factor-like domain that is essential for correct structural folding [13-15]. In particular, MSP5 is highly conserved among *P. falciparum *isolates with a lack of significant antigen diversity, a desirable property for a vaccine candidate [16,17]. For these vaccines to advance to the clinic, they will need to be combined with a suitable vaccine adjuvant, and as part of this project we are screening candidate adjuvants for this purpose.

Part of the difficulty in developing malaria vaccines is the need for a sufficiently potent yet safe human adjuvant to make the vaccine effective. Aluminium hydroxide (alum) or water in oil emulsions, e.g. Montanide ISA720, have been the most commonly used adjuvants in malaria vaccine trials to date. These adjuvants have largely failed to generate protective immunity and in the case of Montanide have proved to be considerably reactogenic [18]. *Plasmodium *antigens may induce a suppressor immune response thereby assisting parasite survival and making it more difficult to induce effective vaccine immunity [19-21]. In animal vaccine challenge studies, complete Freund's adjuvant (CFA) stands out for its ability to induce protective immunity to malaria in situations where alum or Montanide are ineffective.

Although CFA is too toxic for human use, this suggests that the development of an effective malaria vaccine will require more potent human adjuvants than those currently available [22]. To date, greatest success with a malaria vaccine has been seen with RTS,S, a CSP-derived antigen developed by GSK Biologicals in collaboration with the Walter Reed Army Institute of Research. The RTS,S vaccine is formulated in AS02A, a proprietary GSK adjuvant which contains the immune stimulants QS21 and monophosphoryl lipid A in an oil emulsion. In African field trials RTS,S vaccine provided partial short-term protection against malaria and limited disease severity, with the ASO2A adjuvant being potentially at least one factor contributing to the success of this vaccine [23-25].

The importance of the adjuvant selection for malaria vaccine success suggests that the type of the immune response generated by a malaria vaccine may be at least as important as the quantum of the immune response in determining vaccine efficacy. Although rodent models are useful to assess malaria antigen candidates, no small animal models are able to test actual protection against *P. falciparum *challenge [26].

However, a combined humoral and cellular immune response including induction of complement fixing antibodies and cytotoxic T cells against the vaccine antigen is thought to be important for protection against malaria [27-30]. Preclinical studies with recombinant MSP4/5, the single homologue of both MSP4 and MPS5 in *Plasmodium yoelii *(Py), a species of rodent malaria, have shown that it requires combination with a strong Th1 adjuvant such as CFA to provide malaria protection [31]. As CFA is too toxic for human use, as part of the development program for MSP4 and MSP5 as malaria vaccine candidates various adjuvants have been tested for their suitability for inclusion in clinical trials of these vaccines. The development of a novel adjuvant based on the structural transformation into cochleate structures (AFCo1, Adjuvant Finlay Cochleate 1) of proteoliposomes (PL) extracted from the outer membrane of *Neisseria meningitidis *B has been previously reported [32,33]. AFCo1 consists of a highly stable complex of Ca2+, proteins and lipids forming a compact multilayered tubular structure, which can accommodate new antigens from different sources thus allowing its use as an antigen delivery system [32,34,35]. AFCo1 also contain several Neisseria-derived pathogen associated molecular patterns (PAMPs) within its structure, namely porin proteins and lipopolysaccharide (LPS), which powerfully activate the innate and adaptive immune systems and, thereby, provide AFCo1 with strong adjuvant properties [32]. Adjuvant properties of AFCo1 have been previously demonstrated using model antigens, including egg albumin (OVA), *Leishmania majo*r proteins and the gD2 glycoprotein from herpes simplex virus type 2)[36]. These experiments confirmed that AFCo1 induced a potent immune Th1 response characterized by high levels of antigen-specific IgG, class switching from IgG1 to IgG2a, and increased antigen-specific T cells producing IFNγ [37]. Mice immunized with *L. major *antigens plus AFCo1 were protected, suggesting the ability of AFCo1 to modulate the natural induction of a Th2-type responses by *L. major*, resulting in a Th1 switch and a substantial decrease of the progression of lesions after challenge [32].

In the current study, the ability of AFCo1 adjuvant to enhance the immune response against recombinant MSP4 and MSP5 from *P. falciparum *was tested. These studies confirm the utility of AFCo1 to enhance a Th1-type response to incorporated vaccine antigen and this raises the possibility of using AFCo1 as an adjuvant for MSP-based vaccines in future human trials.

## Methods

### Antigens

The full-length MSP4 and MSP5 sequences lacking the secretion signals and the GPI attachment signals were expressed in *E. coli *with a C-terminal hexahistidine tag. The fusion proteins were purified with TALON metal affinity resin according to the manufacturer's instructions. The purify and integrity of the proteins were assessed on SDS-PAGE gels stained with Coomassie blue, and the concentration of the proteins were measured with the Bio-Rad Bradford assay [9,14].

### Adjuvants

Both MSP4 and MSP5 proteins were formulated independently with three adjuvants: incorporated into AFCo1 and Synthetic Cochleate Structure (SCS) or emulsified in CFA; generating six formulations to be tested in animal experiments.

AFCo1 consists of a highly stable complex of lipid membranes from PL and Ca^2+ ^that is formed when PL is slowly exposed to CaCl_2 _buffer solution through a dialysis process. The PL from *N. meningitidis *serogroup B (Cu385, B4:P1.15, 19, L3, 7, 9) used for obtaining AFCo1 was obtained from the vaccine production facility of Finlay Institute, Cuba and contained the major outer membrane proteins (OMP) P1 (Por A), P3 (Por B) and P5 plus other high MW OMP proteins from 65 to 95 KDa, lipopolysaccharide (LPS) (2–6% of total protein content) and bacterial phospholipids as principal components. AFCo1 shows a multilayered tubular structure and conserves all components of PL but in a completely new structure with additional properties [32,35].

SCS shares structural similarities with AFCo1, but instead of being generated from *N. meningitidis *serogroup B PL, it was generated from negatively charged lipid membrane prepared from cholesterol and phosphatidyl serine as detailed below. CFA (Difco, Laboratories, Detroit, MI) was prepared and formulated with antigen following the manufacturer's instructions.

### Antigen incorporation into AFCo1 or SCS

AFCo1 was obtained from PL using a rotary dialysis system as previously described [31]. Briefly, PL was resuspended at 1 mg/mL in Tris (30 mM) – EDTA (3 mM) buffer containing 1.5% sodium deoxicolate (DOC). The solution was pulse sonicated for 5 min in ice and the resulting transparent solution was dialysed 24 h against a buffer containing 5 mM of CaCl_2_. During the dialysis process, the DOC was removed allowing the formation of AFCo1 by the interaction of divalent cations with the lipid membranes of PL. SCS were obtained from lipid vesicles containing phosphatidyl serine and cholesterol (9:1). Briefly, cholesterol was dissolved in a solution of phosphatidyl serine in chloroform, vacuum dried under nitrogen and resuspended in Tris buffer (30 mM). The solution was then dialysed against Ca^2+ ^buffer under the same conditions used for making AFCo-1. MSP4 and MSP5 incorporation into AFCo1 or SCS was performed in independent batches by the same methodology but adding the proteins into the PL or lipid vesicles solutions (1:2 ratio proteins/PL or 1:2 ratio proteins/synthetic liposomes, respectively) before dialysis. The dialysis products were centrifuged and the pellet was then resuspended in dialysis buffer. The incorporation of MSP4 and MSP5 into AFCo1 and SCS was confirmed by SDS-PAGE and western blotting. The efficiency of the incorporation process into AFCo1 or SCS was over 85% for both proteins. Protein concentration was measured by BCA assay kit (Sigma) following the manufacturer's instructions and adjusted for immunization experiments.

### Animal experiments

The immunogenicity of the different formulations containing MSP4 or MSP5 was assessed using female BALB/c mice of 60–100 days of age. Mice were randomly distributed into four groups of 10, and immunized at 14-day intervals with three doses of 10 μg MSPs (all groups received the same amount of MSP): Group 1 was immunized with MSP in PBS, groups 2 and 3 received MSP incorporated into SCS (MSP-SCS) or AFCo1 (MSP-AFCo1) respectively, and group 4 received the MSP emulsified in CFA (MSP-CFA). Delayed-type hypersensitivity reactions (DTH) were assessed 21 days after the last immunization. Serum samples were taken at 21 and 35 days after last immunization. On day 45, mice were sacrificed and spleen cells were obtained for proliferation assays and cytokine determination. MSP4 and MSP5 proteins were studied independently in two different experiments but following the same design as described above.

### Serum antibody measurement

MSP and PL-specific IgG antibodies were determined by standard ELISA protocols. In brief, ELISA plates were coated overnight at 4°C with MSP 2 μg/mL or PL 10 μg/mL. Mouse serum samples in duplicate were used at 1/1000 dilution and incubated in ELISA plates for two hours at room temperature (RT). HRP-conjugated monoclonal antibodies (Pharmingen) were used to detect total antigen-specific IgG, IgG1, or IgG2a. TMB (3,3',5,5'-tetramethylbenzidine) was used for a readout and absorbance read at 450 nm after stopping with 1 M H_2_SO_4_. Titers were expressed as arbitrary units read from an internal lab-derived standard curve.

### Delayed-type hypersensitivity reaction

DTH reactions were assayed against MSP4 or MSP5 independently in the footpad of immunized mice. Antigens were prepared in PBS (0.1 mg/mL) and 50 μL was injected intradermally (5 μg) in the left footpad using a 30 G needles. The same volume of PBS was injected into the right footpad as a control. The presence of footpad reactions was checked at 15 min, 4 h and 24 h. The extent of inflammation in the footpad was measured 48 h after injection using a spring gauge calliper. Antigen-specific DTH was accessed as the mean of the difference of thickness between the left (antigen) and right (PBS) footpad.

### Spleen cell proliferation assay

The induction of memory T and B cells was assessed *in vitro *by proliferation of spleen cells in response to MSP stimulation. Proliferation was detected by dye dilution method of CFSE stained cells, as previously described [35,36]. Briefly, mice were sacrificed by cervical dislocation and the spleens were aseptically removed. Spleen cells were harvested by flushing the spleen with PBS. Red blood cells were eliminated by treatment with a hypertonic solution of NH_4_Cl for three minutes on ice followed by centrifugation and washing. Cells were stained with CFSE (2 μM) for 10 min at 37°C, washed three times with PBS containing 20% foetal calf serum (FCS), and resuspended at 8 × 10^6 ^cells/mL in RPMI-1640 medium supplemented with L-glutamine, pyruvate, gentamicin, penicillin and 10% FCS. Cells were culture in 24-well plates (8 × 10^6 ^cells/well, 2 mL/well) in the presence of MSP (10 μg), PHA (5 μg), hepatitis B surface antigen (5 μg) or medium (background control). After five days of culture, cells were harvested and analysed for proliferation using FACScanto cytometer (Becton Dickinson). Proliferating cell populations were identified by staining with CD4, CD8 or CD19 PE-labelled monoclonal antibodies (Pharmigen). Culture supernatants were collected for cytokine determination.

### Cytokine assay

The production of IFNγ and IL5 by proliferating splenocytes was measured in the culture supernatant by ELISA. Capture and peroxidase-conjugated detection antibodies (Pharmigen) were used according to the manufacturer's instructions. The quantities of each cytokine were determined using standard curves with recombinant IFNγ or IL5.

### Statistical analysis

Descriptive statistical variables (mean and standard deviation) were calculated for all data and presented graphically. Normality of data was analysed using Kolmorgorov-Smirnov normality test and comparisons between groups were accessed by one-way ANOVA followed by a Tukey multiple comparison test. P value of 0.05 was used to determine significant differences.

## Results

### MSP-specific IgG responses and subclass composition

**Figure 1:**
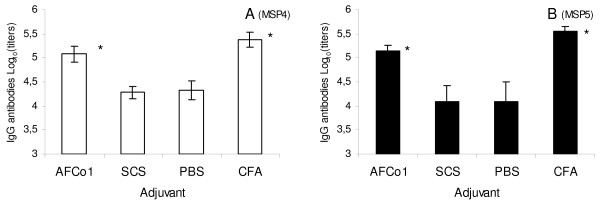
**Influence of adjuvant on anti-MSP4 and MSP5 IgG production**. The adjuvant effect of AFCo1, SCS or CFA on the immune response against MSP4 or MSP5 was explored in separated immunization experiments. Mice were injected with three intramuscular doses at a 14-day interval of MSP4 or MSP5 incorporated into AFCo1 or SCS, or mixed with CFA or PBS. Serum samples were taken 21 days after the last immunization, MSP4 (Panel A) or MSP5 (Panel B)-specific IgG were measured by ELISA. Antibodies levels are expressed as a logarithm of the titer. Each experiment was repeated 3 times. The means and standard deviations are showed. * Significant differences by Tukey test (p < 0.05) compared with PBS group.

**Figure 2:**
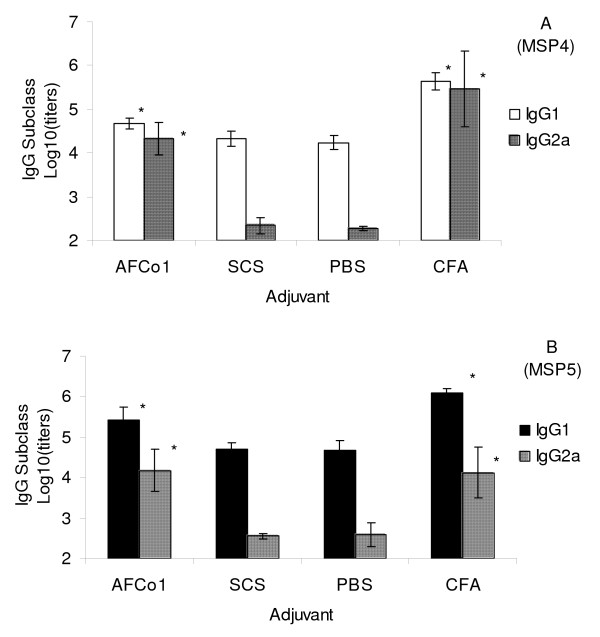
**Influence of adjuvant on anti-MSP4 and MSP5 IgG isotype production**. The adjuvant effect of AFCo1, SCS, CFA or PBS on the immune response against MSP4 or MSP5 was compared. Mice were injected with three intramuscular doses at a 14 day interval of MSP4 or MSP5 incorporated into AFCo1 or SCS, or mixed with CFA or PBS. MSP-specific IgG1 and IgG2a were measured by ELISA in serum taken 21 days after the last immunization. (Panel A) MSP4-specific IgG1 (open bars) and IgG2a (lined bars) from MSP4 immunized mice. (Panel B) MSP5-specific IgG1 (filled bars) and IgG2a (lined bars) from MSP5 immunized mice. Antibodies levels are expressed as a logarithm of the titer. The means and standard deviations of 3 different experiments are shown. * Significant differences by Tukey test (p < 0.05).).

In two separate experiments, mice were immunized intramuscularly with 10 μg of MSP4 or MSP5 adjuvated with CFA, PBS or incorporated into AFCo1 or SCS. Twenty one days after the third and final immunisation, mice injected with MSP in PBS had measurable anti-MSP antibodies in their sera (Figure 1). However, anti-MSP titers were significantly increased when MSP was administrated in CFA or AFCo1 with no significant differences between CFA and AFCo1 induced titers. In contrast, no adjuvant effect was observed when MSP was incorporated into SCS, with antibody titers not significantly different to those seen with MSP in PBS. The IgG subclass composition of the anti-MSP response was highly dependant on the adjuvant used. MSP in PBS or SCS induced exclusively IgG1 antibodies with no measurable IgG2 response, whereas AFCo1 and CFA induced both high IgG2a and IgG1 suggesting a preferential induction of a Th1-type response, similar to CFA which also induced high IgG2a and IgG1 (Figure 2).

### MSP-specific DTH reaction

**Figure 3:**
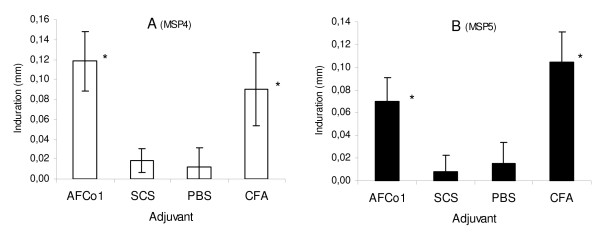
**Delayed type hypersensitivity reaction induced by intradermal challenge with MSP4 or MSP5 in immunized mice**. Mice were immunized with MSP4 or MSP5 incorporated into AFCo1 or SCS, or mixed with CFA or PBS (3 intramuscular doses, 14 day interval). The presence of delayed type hypersensitivity (DTH) was tested 21 days after the last immunization by intradermal challenge with 5 μg of MSP4 or MSP5 in the left footpad. PBS was injected in the right footpad as control. The size of inflammation in both footpaths was measured 48 h after challenge using a caliper. The induration induced by the antigen was measured as the difference of right minus left footpad thickness. The magnitude of DTH reaction induced by MSP4 (Panel A) and MSP5 (Panel B) immunization are expressed as the mean and standard deviation of the induration in each experimental group. * Significant differences by Tukey test (p < 0.05).

The development of DTH skin reactions 48–72 hours after intradermal injection of a sub-immunizing dose of antigen provides evidence of a cell-mediated or Th1 response. Based on its ability to induce IgG2a, AFCo1 may induce a Th1-type immune response. To confirm this possibility, DTH reactions against MSP were assessed in all experimental groups. As shown in Figure 3, groups immunized with MSP4 or MSP5 in SCS or PBS did not develop a detectable DTH reaction following intradermal challenge with MSP4 or MSP5. In contrast, groups immunized with MSP incorporated into AFCo1 or adjuvated with CFA developed strong DTH responses after intradermal challenge with MSP, in agreement with the increased levels of MSP-specific IgG2a detected after immunization. The size of the DTH reaction in mice receiving the AFCo1-MSP formulations was significantly higher than that in the CFA-MSP injected group.

### MSP-specific splenocyte proliferation

**Figure 4:**
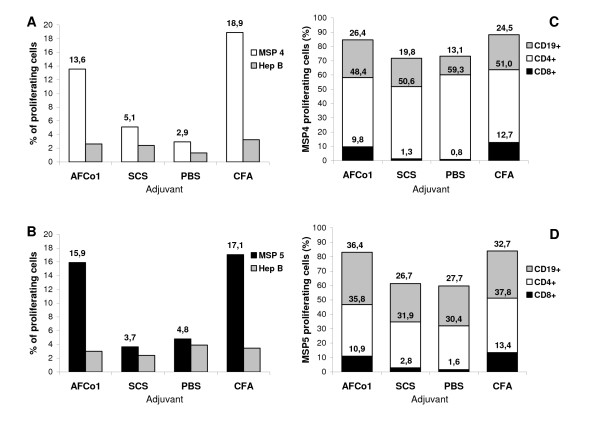
**Influence of adjuvant on MSP4 or MSP5-induced splenocyte proliferation**. Splenocytes from mice immunized with MSP4 or MSP5 in different adjuvants were isolated, stained with CFSE and cultured in the presence of MSP4 or MSP5 at 10 μg/ml. HBsAg was used as an unrelated antigen control. PHA and medium alone were used as positive and negative controls respectively (data not show). After 5 days, proliferation was detected by the presence of cells with a decreased fluorescence intensity compared with the negative control (medium control). The frequencies of MSP4 (Panel A) or MSP5 (Panel B) specific proliferating cells are expressed as percentages of the total population. Proliferating populations were identified and quantified by gating specific proliferating cells previously stained with anti-CD4, CD8 or CD19 PE labeled monoclonal antibodies. The distribution of proliferating populations is showed as percentages of the total number of MSP4 (Panel C) or MSP5 (Panel D) stimulated proliferating cells.

CSFE-based spleen cell proliferation assays to MSP were performed to compare the extent and type of memory T-cell responses induced by each adjuvant. Mice were sacrificed 45 days after the final immunization and splenocytes isolated and cultured for 5 days in the presence of MSP, hepatitis B surface antigen (HBsAg) (control antigen), PHA (control mitogen) or medium alone. Proliferation was only observed when splenocytes were stimulated *in vitro *with MSPs or PHA (Figure 4A, Figure 4B) and as expected no proliferation of splenocyteswas induced by medium alone or HBsAg control (Figure 4A, Figure 4B). Splenocytes from mice immunized with AFCo1-MSP or CFA-MSP showed significantly higher proliferation than splenocytes from SCS-MSP or PBS-MSP immunized mice. Although the percentage of proliferating CD4+ T cells was similar in all groups, differences were detected in CD8+ T-cell proliferation between adjuvant groups (Figure 4C, Figure 4D). CFA-MSP and AFCo1-MSP immunized mice showed significantly increased CD8+ T-cell proliferation to MSP over SCS-MSP or PBS-MSP immunized groups. Consistent with the higher levels of MSP-specific IgG antibodies in the AFCo1 or CFA-adjuvanted MSP groups, there was an increased frequency of B-cell proliferation to MSP in these groups. Comparing the MSP4 and MSP5 experiments, similar results between groups were observed with all immunized groups having high CD4+ T cell proliferation to MSP whereas only the CFA-MSP and AFCo1-MSP groups had significantly increased CD8+ T-cell and B-cell proliferation to MSP.

### MSP-specific cytokine production

**Figure 5:**
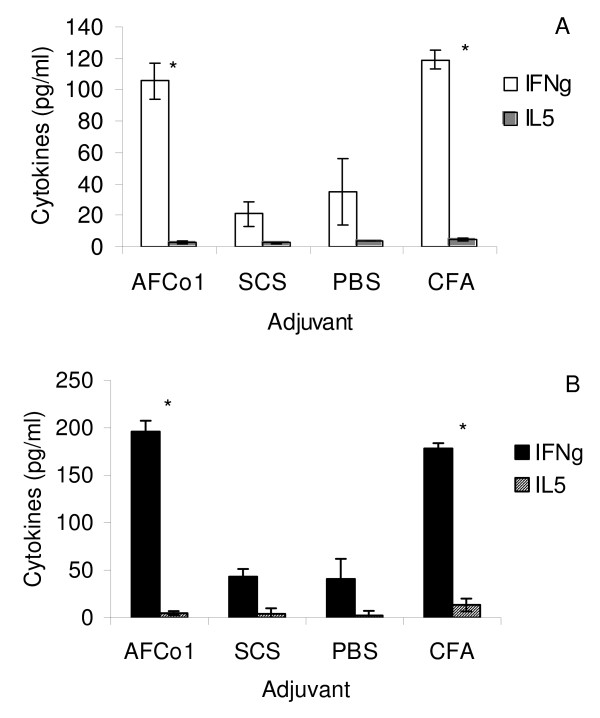
**Influence of adjuvant on MSP4 or MSP5-induced cytokine production**. Splenocytes from mice immunized with MSP4 or MSP5 in different adjuvants were cultured in the presence of MSP4 or MSP5 at 10 μg/ml. After 5 days of culture, supernatants were colleted and analysed for the presence of IFNγ or IL5 using a capture sandwich ELISA. Panel A shows the levels of IFNγ and IL5 produced by spleen cells from mice immunized with MSP4 in AFCo1, SCS, PBS or CFA after *in vitro *recall with MSP4; whereas panel B shows the levels of IFNγ and IL5 produced by spleen cells from mice immunized with MSP5 in AFCo1, SCS, PBS or CFA after *in vitro *recall with MSP5. The means and standard errors are shown. * Significant differences by Tukey test (p < 0.05).

MSP-specific cytokine production (IFNγ and IL5) was measured in the culture supernatants of splenocytes incubated with MSP for 5 days. Splenocytes from mice immunized with CFA-MSP or AFCo1-MSP produced significantly higher levels of the Th1 cytokine IFNγ in response to MSP stimulation than splenocytes from SCS-MSP and PBS-MSP injected mice (Figure 5). MSP-stimulated IL5 production was low in all groups.

## Discussion

The results show that AFCo1 is an effective adjuvant that induces a strong antibody and T-cell immune response to MSP4 or MSP5 vaccine antigens with potency comparable to CFA. AFCo1 stimulated high levels of MSP-specific IgG2a, IFNγ, and CD4+ and CD8+ proliferation consistent with induction of a Th1-type immune response. In addition, only mice immunized with AFCo1-MSP or CFA-MSP developed a DTH reaction following intradermal challenge with MSP. How relevant these observations are to malaria protection remains to be confirmed in a challenge model. Unfortunately, only *in vitro *parasite infectivity growth inhibition assays are available for *P. falciparum*. Thus no definite *in vivo *correlates of protection are available for human malaria. However, *Stewart et al *similarly evaluated the adjuvant effect of different formulations of RTS,S vaccine by DTH and cellular outcomes and suggested that a Th1 response could be a good marker of effectiveness for adjuvant suitability for malaria vaccine development [38,39].

Considering the complexity of *Plasmodium *life cycle, a protective immune response should probably include multiple mechanisms to target different stages. Increased CD8 T-cell activity against *Plasmodium *antigens may help eradicate the intracellular stages of the *Plasmodium *human cycle by mechanisms including cross-presentation of parasite-derived antigens [40,41]. On the other hand, immunological studies in malaria patients suggest that the level of antibodies against blood stages of *Plasmodium*, predicts recovery from malaria. In addition, *in vitro *infection inhibition studies have demonstrated that antibodies directed against MSP1 inhibit parasite infectivity and red blood cell (RBC) invasion [42], suggesting that MSP proteins could be promising candidates for vaccine development [11,30,43]. Even the role of antibody in malaria protection remains uncertain; some functional properties of antibodies like specificity, subclass and binding affinity are likely to be important for parasite elimination.

A combination of antibody-dependent and cell-mediated mechanisms may provide the most efficient immunity against *Plasmodium *infection. Even although antibodies may be effective against circulating parasites, a T-cell response is also needed to eliminate intracellular parasites [44-47]. A Th1 type immune response should be beneficial given the need for complement fixing antibodies and CTL activity to completely eliminate intracellular parasites [40]. The induction of a Th1 immune response could provide an adequate environment for the induction of such mechanisms. The presence of high levels of IFNγ drive the production of IgG subclasses with high complement binding properties like IgG2a, as well as the differentiation and proliferation of CD8+ T cell with potential CTL activity.

The ability of AFCo1 to induce a Th1-like response has been previously demonstrated using different antigens, including the ability to induce a switch from a Th2 to Th1 response [32]. Strong CD4+ T-cell proliferation and high levels of IFNγ production upon *in vitro *stimulation of spleen cells from immunized mice further supports the Th1 polarizing capability of AFCo1. The results from the present experiments, confirmed the Th1 polarizing properties of AFCo1 but using MSPs antigens from *P. falciparum*. This property is comparable with the adjuvant action of CFA, since only animals from groups receiving MSP in CFA or AFCo1, showed similar results on the Th1 measuring tests.

The Th1 adjuvant properties of AFCo1 could be explained by the presence of strong activators of the immune system within from the MBOMP like LPS and Porins. These components are present in the PL and are conserved during its transformation into AFCo1. LPS and Porins from B meningococcus are well known activators of TLR signalling pathways on dendritic cells and conditioning Th1 immune response polarization.

This possibility could be further supported from the comparison of the immune response induced by AFCo1 and SCS. The use of synthetic cochleates as a control showed that the SCS sharing a similar cochleate structure with AFCo1, had no adjuvant effect as anti-MSP IgG levels were no different as to when the antigen was given in PBS. This suggests that the adjuvant effect of AFCo1 is highly dependent on the PAMPs from *Neisseria *rather than the cochleate structure per se. Furthermore, it could also support the potential use of bacterial-derived adjuvants for malaria vaccines development [48,49]. In fact, the two successful adjuvants in this study, AFCo1 and CFA, both contain components derived from bacterial membranes that are able to provide strong immune activation signals [50,51]. Notably, the CFA-MSP and AFCo1-MSP groups also showed the highest level of CD8+ T-cell proliferation to MSP.

Recent data also suggest the ability of parasites to induce immune regulatory responses during infection that inhibit host immune defence mechanisms, which adds another barrier to the development of malaria vaccines [19]. The presence of Foxp3, CD25+CD4+ T cells has been reported to correlate with more rapid parasite growth during *P. falciparurm *infection [20]. Regulatory T cells block the activation of parasite elimination mechanisms and enable the parasite to escape from host immunity [19,44]. Thus, an ideal adjuvant for use with malaria vaccines must be capable of inducing a strong Th1-type response and overcome parasite-induced regulatory mechanisms. The ability of AFCo1 to specifically abrogate the regulatory mechanisms induced by *Plasmodium *remains to be further explored. In most studies that have shown protection against parasite challenge, CFA was the adjuvant able to generate the best protection. Immunisation with MSP4/5 or combinations of MSP4/5 and MSP1, in CFA induced a high level of immunity that protected mice from lethal challenge [31,52-54]. Although, no *P. falciparurm *animals challenge models closely approximate human infection, the fact that CFA is able to induce protective immune response to *P. yoelii *in mice suggests that a human adjuvant of equivalent potency may be required to achieve a successful malaria vaccine.

## Conclusion

Considering that CFA induces protective response in animal models of malaria challenge, the fact that AFCo1 and CFA induce a similar immune response to MSP in mice suggests the potential of AFCo1 as a potent adjuvant for human malaria vaccines, particularly given that the constituents of AFCo1 are already approved for human vaccine use.

## Competing interests

The authors declare that they have no competing interests.

## Authors' contributions

GB made the adjuvant, undertook the mouse experiments and with NP conceived the project and study design, CZ assisted with mouse experiments, LW and RC contributed the MSP protein constructs and participated in study design, OP participated in study design and NP coordinated the project including study design and execution and manuscript preparation. All authors read and approved the final manuscript.
